# Echolocating bats rapidly adjust their mouth gape to control spatial acquisition when scanning a target

**DOI:** 10.1186/s12915-022-01487-w

**Published:** 2022-12-17

**Authors:** Ofri Eitan, Mor Taub, Arjan Boonman, Amir Zviran, Vladimir Tourbabin, Anthony J. Weiss, Yossi Yovel

**Affiliations:** 1 School of Zoology, Faculty of Life Sciences, Tel Aviv University, 6997801 Tel Aviv, Israel; 2 The School of Electrical Engineering, the Iby and Aladar Fleischman Faculty of Engineering, Tel Aviv University, 6997801 Tel Aviv, Israel; 3 Department of Electrical and Computer Engineering, Ben-Gurion University of the Negev, Beer Sheva, Israel; 4 Sagol School of Neuroscience, Tel Aviv University, 6997801 Tel Aviv, Israel; 5 School of Mechanical Engineering, the Iby and Aladar Fleischman Faculty of Engineering, Tel Aviv University, 6997801 Tel Aviv, Israel; 6 The Steinhardt Museum of Natural History, National Research Center for Biodiversity Studies, Tel-Aviv University, Tel Aviv, Israel

**Keywords:** Bats, Echolocation, Sensory acquisition, Piston model, Mouth gape, 3D Tracking, 3D acoustic simulation, CT scan

## Abstract

**Background:**

As well known to any photographer, controlling the “field of view” offers an extremely powerful mechanism by which to adjust target acquisition. Only a few natural sensory systems can actively control their field of view (e.g., dolphins, whales, and bats). Bats are known for their active sensing abilities and modify their echolocation signals by actively controlling their spectral and temporal characteristics. Less is known about bats’ ability to actively modify their bio-sonar field of view.

**Results:**

We show that *Pipistrellus kuhlii* bats rapidly narrow their sensory field of view (i.e., their bio-sonar beam) when scanning a target. On-target vertical sonar beams were twofold narrower than off-target beams. Continuous measurements of the mouth gape of free-flying bats revealed that they control their bio-sonar beam by a ~3.6 mm widening of their mouth gape: namely, bats open their mouth to narrow the beam and vice versa.

**Conclusions:**

Bats actively and rapidly control their echolocation vertical beam width by modifying their mouth gape. We hypothesize that narrowing their vertical beam narrows the zone of ensonification when estimating the elevation of a target. In other words, bats open their mouth to improve sensory localization.

**Supplementary Information:**

The online version contains supplementary material available at 10.1186/s12915-022-01487-w.

## Background

Echolocating bats are renowned for their ability to control the sensory information they acquire. Through rapid changes in their echolocation signal design and timing, bats adjust, within a few dozen milliseconds, the rate and accuracy with which they acquire information about the environment [1, 2]. Because this degree of control can be documented simply by recording bat audio emissions with a single microphone, decades of research have generated a good understanding of when and how bats control their signal design and timing [3–8]. Much less is understood about bats’ ability to control the spatial characteristics of their emission, that is, their ability to control the sector of space that they scan using a single emission (often referred to as “beam forming”). The bio-sonar beam can be thought of as the bat’s acoustic sensory field of view. Narrowing it, therefore, might have several advantages [9], such as (1) reducing unwanted echoes returning from the periphery; (2) improving angular resolution by directing more energy to a narrower angular sector in space; the width of the beam is the first factor determining echolocation sensory resolution as it delineates the borders of the sector where the reflector might be (see more in the “Discussion” section); and (3) increasing both the absolute echo strength and the signal-to-noise ratio (SNR) by focusing more energy ahead. Widening the beam, in contrast, might be desirable when a wider sector of the environment requires searching.

The introduction of large-multi-microphone-array recordings in the past two decades has allowed researchers to study the bats’ control of their beam-width [10–18]. Several studies have shown that bats dynamically adjust their beam-width to adapt it to the sensory needs of a particular task, for example by widening the beam in the last few dozen milliseconds before intercepting prey [15, 17, 19, 20]. How bats do this remains poorly understood. Based on acoustic theory, bats could adjust their beam-width by either changing the spectrum of their signals or the shape of their emitter, essentially their mouth gape (hereafter “gape”) [15, 20]. Previous studies have suggested that bats adjust the spectral content of their signals to control beam-width [19, 20]. The alternative hypothesis—that bats control their beam by altering their gape—has been poorly studied, because changes in mouth gape are on the mm-scale and are extremely difficult to measure in freely-behaving bats [15, 21]. Recently, we used still images and machine-learning to show that Vespertilionid bats control their acoustic beam-width by altering the size of their aperture (i.e., their gape [22]). However, because these were still images, we could not monitor the size of the emitter continuously and thus could not assess the bats’ level of control of this mechanism. Consequently, we were unable to determine in which situations bats use their gape to control beam-width, or how fast they are able to do so.

In this study, we sought to directly assess bats’ control of their gape. We therefore tracked the mouth gape of freely-flying *Pipistrellus kuhlii* bats while performing a search and landing task in the lab. We reveal how bats widen their gape, probably to control beam-width, when they scan a potential target. Using a 3D acoustic simulation, we show that the bats thereby substantially narrow their zone of ensonification. We also demonstrate that the well-studied sensory behavior of a bat approaching landing (i.e., echolocation approach behavior) previously described in terms of signal design and timing, also includes rapid adjustments of gape and, accordingly, of beam-width and zone of acquisition.

## Results

**Fig. 1:**
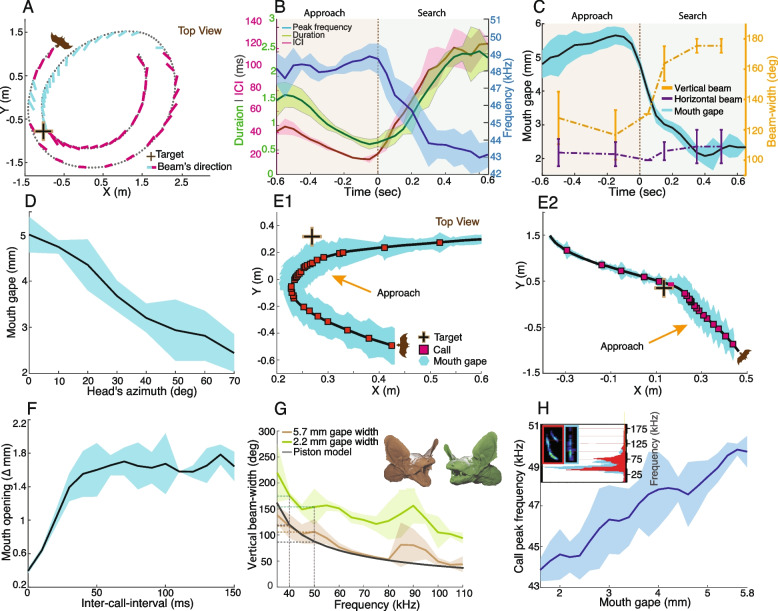
Bats adjust their mouth gape to control acquisition. **A** A bat circling the target (“+”). Flight trajectory—black dotted line; beam direction—turquoise (approach beams), magenta (search beams), flight start and direction depicts by bat symbol. The bat performs two approaches in this trial: an initial one with three beams pointed at the target; and a long one, without landing. **B** Bats increased emission rate, decreased call duration, and increased call peak frequency prior to landing or attempting to land on the target. In both “**B**” and “**C**,” the sequence presents two phases—approach and search. Time “0” depicts the first call after the minimal ICI, mean ± SE, *n* = 5 bats. **C** The bats controlled their vertical beam-width by widening the gape prior to landing. Simulated vertical (yellow) and horizontal (purple) beam widths. Simulations are averaged for two models based on the average gape of all five bats. **D** The bats widened their gape when aiming their gaze (head) at the target (*X* axis shows azimuth relative to target), mean ± SE, *n* = 5. **E1**–**E2** Schematics illustrating two approach trials. Flight trajectory—black line; echolocation calls—red squares; mouth gape—turquoise shading. **F** The change in gape as a function of the ICI, mean ± SE, *n* = 5 bats. **G** 3D acoustic simulation of the vertical beam-width (two-sided 6 dB) as a function of the emission frequency for the observed two gapes: 2.2 mm—green and 5.7 mm—brown. The average of two models is presented. A piston model of a 7 mm aperture is shown in black. Horizontal and vertical dashed lines depict the bats’ peak frequencies during search and approach, respectively (40 and 50 kHz). **H** Call peak frequency increased during the approach. Insert shows two call spectrograms and spectra—search in red and approach in turquoise

Bats were trained to search for the landing platform and land on it for a reward of a mealworm (Additional file 1: Fig. S1, Methods). When flying in the flight room (each bat individually), bats first searched for the target and, upon detecting it, they typically circled it a few times before landing on it (Fig. 1A). While circling, the bats often initiated typical echolocation approach behavior without landing.

As previously described in the literature [19], approach events were characterized by the following: (1) significantly shorter call durations, from 2.3 ± 0.4 ms to 0.5 ± 0.09 ms for search vs. approach calls, respectively (mean ± SE hereafter, Fig. 1B, green line, *P* = 3.97e−10, mixed-effect GLM with duration set as the explanatory parameter, time from landing as a fixed factor, and the individual bats as a random effect; bats decreased their call duration by an average of 0.1 ms/s during the approach, *n* = 5 bats; (2) significantly shorter inter-call intervals (ICI), from 117.9 ± 4.6 ms to 13.6 ± 3.1 ms (Fig. 1B, magenta line, *P* = 6.78e−21, GLM as above with ICI set as the explanatory parameter; bats reduced ICI by an average of 83 ms/s during the approach, *n* = 5 bats; and (3) significantly elevating the calls’ peak-energy frequency (i.e., the frequency with the maximal intensity in the spectrum), from 43 ± 0.9 kHz to 49.4 ± 0.8 kHz (Fig. 1B, blue line, *P* = 4.61e−21, GLM as above with frequency as the explanatory parameter). Bats increased peak frequency by an average of 5.61 kHz/s, *n* = 5 (see statistics and Additional file 2: Table S1 for individual statistics). In all of the above analyses, the trials were aligned relative to time “0,” defined as the time of the first call after the minimal ICI along the sequence. We defined the beginning of the approach phase as the moment when the ICI dropped to below 40 ms.

With some of the bats (*n* = 5, Methods), we used a microphone array to estimate the horizontal direction of the beam and found that during these approach events the bats directed their beams towards the target, steering the beam by positioning their head accordingly (see Methods, Fig. 1A and Additional file 3: Fig. S2). During searching, the bats directed their beams in the direction of flight, with a slight bias towards the turning direction (Additional file 4: Fig. S3).

Continuous tracking of the bats’ gape, using a high-accuracy, high-frame rate (300 fps) multi-camera tracking system, revealed that the bats also significantly widened their mouth gape during the approach. When directing their beam at the target, the bats widened it from an average of 2 ± 0.2 mm during the search phase to 5.6 ± 0.2 mm during the approach (Fig. 1C–E, *n* = 5 bats, *P* = 2.35e−30, mixed-effect GLM with gape set as the explanatory parameter, time as a fixed factor, and the individual bat as a random effect; the mouth opening rate was 3.3 mm per second, *n* = 5 bats, see Statistics).

During the search phase, the bats re-opened their mouth with each emission and then closed it; whereas during the final part of the approach, their mouth remained almost completely open (with small modifications, Fig. 1F). This suggests that when the ICI drops below ~40 ms, the bats leave their mouth open, probably due to the difficulty of performing such rapid gape adjustments (Fig. 1F). We estimated the speed of mouth opening and found that it was generally constant, reaching 90.7 ± 8 mm/s independently of the behavioral phase (*n* = 5, Additional file 5: Fig. S4).

We next used numerical acoustic simulations based on the boundary element model (BEM) method, which estimates the form of an acoustic beam given a complex emitter shape [16, 23, 24] (Methods). The simulations utilized a geometrical model obtained through a high-resolution 3D CT scan of a *P. kuhlii* bat and estimated the expected (horizontal and vertical) beam-width when its mouth was open or closed (the degree of opening was based on our observations on the real bats, Fig. 1C). Simulations showed that opening the mouth to the extent that our bats did, should have a dramatic effect on the vertical beam-width, narrowing it by 53 ± 12.8° (Fig. 1G; estimated around the bats’ peak frequency, we used the mean of beams at 40 and 50 kHz). Furthermore, because the bats also elevate their peak frequency during the approach (Fig. 1H), the beam is further narrowed by another 20.6 ± 13.9° when measured at its peak frequency (Fig. 1C, G). The horizontal beam did not change (Fig. 1C and Additional file 6: Fig. S5).

## Discussion

By altering their mouth gape, echolocating bats rapidly narrow the zone of ensonification, and thus control their sensory spatial acquisition when focusing their acoustic attention on landing on a potential target. By opening their mouth, bats in our experiment narrowed their vertical beam-width by ca. 53° upon directing their sensory gaze towards a target. This ~50° narrowing occurred at all beam frequencies (Fig. 1C, G). Elevating the peak frequency further narrowed the beam by another ca. 20°, accounting for a total narrowing of the approach beam by ca. 50% at its most intense frequency in comparison to the search-phase beam.

The function of this behavior in intriguing. Narrowing the beam improves angular accuracy, enabling scanning of a more limited sector, reducing the uncertainty in estimating the position of a reflecting object, and in this case improving the accuracy of estimating the target’s elevation [25]. While accurate horizontal (azimuth) localization can be achieved using inter-aural time and intensity differences, vertical angular localization (elevation) is much less accurate [26, 27] and is probably substantially improved by the observed gape adjustment. Narrowing the beam should also improve the SNR of the target, because more energy is focused on-axis and thus reflected from the target, and fewer echoes are reflected from background objects in the room.

The narrowing of the beam accompanies the well-documented echolocation approach-phase, which has been studied in numerous previous works and is characterized by a sequence of signals that stereotypically change in duration and frequency as a function of the distance to the target [3, 28–31]. In this study, we show that the approach phase also includes spatial sensory dynamics: namely, a change in the beam-width. The bats also narrowed their beam when directing it towards the target even when not performing a full approach—merely a brief, aborted approach (such approach initiations were sometimes as short as 3-signals). These events (which occurred almost every time the bat passed the target, see example in Fig. 1A) were characterized by typical approach signal adjustments (e.g., reducing signal duration), which we interpret as estimations of the target’s position prior to landing or initiation of an aborted approach.

Our results demonstrate that gape-controlled beam adjustments are part of a repertoire of mechanisms that bats routinely apply to control their active sensing. This study provides new insights into our previous finding that bats narrowed their beam to avoid undesirable background echoes when flying through a narrow passage [22]. Their speed of lip-movement was ca. 90 mm/s and during search the bats consistently closed and opened their mouth (Additional file 5: Fig. S4). During the last part of the approach, when the bats emit a series of calls at short intervals, their mouth remains constantly open, probably due to the physical difficulty of rapidly altering the gape under such high emission rate. Our gape estimates are in accordance with anatomical and acoustic estimates performed on a similar-sized species from the same family [15].

In a previous study, Jakobsen et al. [20] showed a widening of the beam via lowering call frequency during the final few dozen milliseconds before reaching the target (also known as the terminal buzz). We show here that from the beginning of the approach-phase (several hundred milliseconds before reaching the target) bats widen their mouth gape and leave it open, thus narrowing their beam (Fig. 1C, F). From a functional point of view, this is reasonable: the initial narrowing allows better SNR and localization when planning the approach, while the final widening during the last few milliseconds is suggested to enable tracking escaping insects just prior to interception (where any slight movement results in large angular shifts). It remains an open question as to whether bats also narrow their beam at the beginning of an insect interception maneuver.

We found that the beam formed by a bat model with a 5.7-mm gape most closely fitted a circular piston model with a 7 mm diameter (Fig. 1G). In other words, the bat’s beam is narrower than that of a piston for a given diameter. One reason for this may be that the bat's mouth is a resonator that creates a pressure distribution inside the aperture (gape-width) that is narrower than the first order Bessel function of the circular aperture piston model. Alternatively, the difference might be a result of a measurement error in our estimation of the exact bat gape (i.e., the actual gape might have been 1 mm wider than we estimated).

## Conclusions

In this study, we quantified the continuous dynamic changes in mouth gape of free-flying echolocating bats, i.e., their control of the echolocation beam-width. We found that by widening their gape bats narrow their vertical beam width and reduce the zone of ensonification. The bats rapidly adjusted their gape according to the particular task they were performing, suggesting that this constitutes another tool in the bats’ active sensing toolbox.

## Methods

### Animals

Ten *P. kuhlii* bats were captured under permit from the Israeli National Park Authority (permit no. 2016/41421). They were housed at Tel Aviv University’s Zoological Gardens under a reversed light-dark cycle and a temperature of 23–26°C. Experimental protocols and procedures were approved and performed according to the Institutional Animal Care and Use Committee of the Israel Ministry of Health (Ethics permit: 04-18-026).

### Experimental setup and training

The flight room where the bats were flown comprised a 5.5 × 4.5 × 2.5 m^3^ room with acoustic foam on the walls and ceiling (Additional file 1: Fig. S1). Audio recordings were made using 46 ultrasonic wide-band microphones (CM16) connected to four Hm1216 AD converters (Avisoft Bioacoustics), synchronized by injecting an SMPTE code (Horita) into the least significant bit of their first channel. The microphones were evenly spread in four rows around the perimeter of the room at 100 cm intervals and at heights of 0 cm, 60 cm, 120 cm, and 180 cm. The bats were trained to search for and land on a platform where mealworms were offered, except during the mouth-gape assessment experiments, in which there were no mealworms on the plate in order to prevent the bats from chewing and interfering with data collection. The landing platform was a 15 cm diameter styrofoam sphere mounted on a ca. 100 cm high pole.

A total of ten bats took part in the experiments, five in the gape tracking experiments and five in beam reconstruction, in order to establish the relationship between the direction of the head and the beam (see below). The data obtained from the latter are presented in Additional files 3 and 4: Figs. S2-S3 reveal a very strong correlation between the direction of the “beam” and that of the head (*R* = 0.91, Pearson correlation). Panel A in Fig. 1 presents the data from one of these bats.

### Tracking

Tracking was performed using a Motion - Analysis Corp system. Sixteen cameras (12 Raptor 1280 × 1024-pixel cameras and 4 Raptor-12 4096 × 3072-pixel cameras) tracked the bats at a frame rate of 300 fps to a spatial accuracy of less than 1 mm. We confirmed experimentally that the system was able to track a moving reflector with an accuracy of ~1 mm and to detect movements as small as 2 mm between two markers (see paragraph below and [32]). Spherical reflectors were glued to the target (6 mm marker) and to the bats using skin bond latex cement (OTSO-BOND Montreal Ostomy Corp.). Two reflectors (1.6 mm diameter, 3X3 Designs Corp.) were glued on either side of the bat’s mouth, and two reflectors (2.4mm diameter, 3X3 Designs Corp.) were mounted on the center of the bat’s head in a cross-shape to enable tracking the head azimuth. Head azimuth relative to a target was defined as the angle between the horizontal direction vector of the head and the vector from the head to the target. The distance between the two mouth markers was adjusted by subtracting a bias of ~5mm—which was the additional distance we added due to our positioning of the markers slightly above and below the edge of the lip (we measured the bias for each bat when the mouth is closed and subtracted the exact individual bias from the measurement results). Even though the echolocation signal can be as short as ~1 ms, tracking the mouth at 300 fps is sufficient to monitor the changes in mouth gape because the lip movement is at least an order of magnitude slower (i.e., the period of a mouth opening cycle is in the order of 40 ms, as we validated from 800 fps videoing, Additional file 7: Movie S1).

### Synchronization of movement and audio

The tracking system included a synchronized audio channel. The microphone connected to this channel was mounted on the platform next to one of the CM16 microphones (Avisoft Bioacoustics). Cross-correlating the two channels allowed us to synchronize the two systems to an accuracy of < 1 ms [32].

### Audio analysis

#### Detection

The echolocation calls were detected and marked using a custom-made MATLAB-based software “batalef” [32, 33]; they were then manually scrutinized.

#### Selection of mouth gape related calls

For the acoustic analysis of the gape condition we chose, for each beam, the microphone with the loudest calls; hence, the highest SNR and an approximation of an on-axis call. We then extracted for each call its ICI (inter-call-interval—defined as the time between the start of one call to the start of the following call in a sequence), duration (defined by the call segment in which the envelope drops by 12 dB relative to the peak), and the peak frequency (the frequency with most energy in the spectrum).

#### Mouth-gape analysis

The gape was measured during call emission for each individual bat. Flight sequences in which the gape was measured were divided into approach and search phases according to an ICI of either more or less than 40 ms, respectively. In most analyses, we used trials that included both search and approach phases (totaling 45 trials for all 5 bats). For some analyses (i.e., head azimuth (Fig. 1D), mouth opening and speed (Fig. 1F and Additional file 5: Fig. S4) and call peak frequency (Fig. 1H)), we added trials incorporating only search behavior (a total of 23 additional trials for all bats).

#### Controlling for the tracking system accuracy

The accuracy of the tracking can be finer than the size of the marker (e.g., a < 1-mm tracking accuracy can be achieved with a 1.6-mm diameter marker), due to the system tracking the center of the spherical marker (as confirmed experimentally below). Therefore, as long as the two markers do not come close enough to be confused by the system, the size of the marker does not limit the tracking accuracy. This allowed us to track two markers as close as 6–7 mm apart (one on either side of the lips) using 1.6 mm markers. The system has already been used in several published studies for tracking movement on similar scales (e.g., [32, 34]). However, to validate its accuracy, we ran a series of control experiments to ensure that our system was able to measure movements in the order of a few millimeters (see Supplementary Figure 1 in Eitan et al, 2019 [32]). In our control experiments (previously published), we measured the position of a stationary marker in different locations in the flight room, with the position tracked over time to estimate the jitter (the standard deviation of the position). In addition, to assess possible error in estimating the distance between two stationary markers, we placed a pair of markers ~5 mm apart at 10 different positions in the room and tracked them over time. Next, to test the robustness of tracking the distance between two markers on a moving object, the same two markers were placed on the pendulum of a mechanical metronome (Wittner) which was moved around the room, while the two markers remained stationary relative to one another (~5 mm apart). This was repeated in nine positions in the room. Because, in this study, we were tracking two markers moving relative to each other (the lips) on a flying bat, we ran an additional control experiment (Additional file 8: Fig. S6). We placed one marker (1.6 mm diameter) on the bottom of the pendulum and another on the fixed base of the metronome (7 mm apart). We first estimated the distance between these two markers while the metronome was stationary and then while it was moved through the room. We repeated this for 10 positions in the room and the estimated error was in the order of 0.06 mm (Additional file 8: Fig. S6). All of the above controls were performed with the same camera setup and frame-rate as in the experiments. Finally, to demonstrate the performance of the tracking system, we provide a film showing the tracking on a metronome pendulum (Additional file 9: Movie S2).

#### Estimating beam direction

We reconstructed 2D emission beams to assess the bat’s acoustic gaze. An 8-ms window was automatically defined around each peak and its spectrogram was estimated (using an FFT window of 1,024 samples with a flat-top window of 512 samples and an overlap of 480 samples). Any spectral content outside the main frequency range of the bat (35 kHz to 90 kHz) was also nullified. By applying MATLAB’s medfreq() function to the spectrogram (after thresholding), we obtained our primary assessment of the signal’s ridge—a frequency over time vector representing the center of the signal in the spectrogram. This function estimates the median frequency of the spectrogram for each time sample. Based on our preliminary knowledge of the downward chirp-like shape of the bat’s calls, we searched for the start of a monotonic decrease in the above function’s output that marked the beginning of a call. The call was terminated either when reaching the end of the ridge (i.e., a 6 dB decrease from its peak) or when its frequency started climbing, suggesting that it had reached the start of an echo. This procedure was iterated several times.

Following estimation of the signal’s ridge, we were able to estimate signal intensity for each microphone and each frequency. Finally, based on the location of the bat when the signal was emitted, we compensated for both the spread loss and air attenuation and calculated the position of each of the microphones relative to the bat during the emission, which provided the samples used later in the beam reconstruction process. Due to the limited 3D spread of the microphones, we only reconstructed the 2D cross-section of the beam. Next, the direction of each beam was obtained by fitting the samples (recorded in all channels) to a Gaussian beam model and taking its maximum as the direction of the emission. Any beam that did not present a good fit to this model (i.e., the R-square in the least mean square analysis was lower than 0.7) was excluded. This procedure was aimed at removing low-quality beams and resulted in the exclusion of ~ 15% of the data.

#### Boundary element model simulation

Employing a previously used method [35], we CT scanned (VECTor4 CT, MILabs) five deceased *P. kuhlii* individuals (the best preserved, with one having died less than 12 h earlier). The bat’s mouth was open during the scan and, based on the data obtained, the two bats with the best scans were selected. The two selected scans were converted into a triangular stl mesh using Amira 6.2.0 (Thermo Fisher Scientific) and down-sampled using 3dsMax (Autodesk) and Meshmixer (Autodesk). The bat’s internal mouth and lips had the highest mesh resolution in the model (0.1 mm; 30-fold shorter than the shortest wavelengths used), and only this part was shown to contribute significantly to the beam. Mesh edge length was increased progressively to 1.2 mm on the exterior features, which had their normals directed away from the measurement area.

The triangular mesh was verified to be closed in on itself, that is, to have no holes, no non-manifold vertices, and with all faces being coherent. We used BEMFA (boundary element modeling) to calculate the beam emitted from the bat’s mouth at a distance of 0.5 m using 8281 measurement points spaced 2° apart from − 90 to 90 °, vertically and horizontally. We verified that pressure generated by the 1 Pa source inside the rear of the bat’s mouth did not leak into the head itself (which could happen if the mesh was faulty). Simulations of the sound field emanating from the mouth were performed at frequencies from 35 to 110 kHz with steps of 5 kHz. We highlighted the bats’ peak signal frequencies during search and approach, respectively (40 and 50 kHz). In each simulation, the Helmholtz equation simulating the complex sound pressure inside the scene, with the entire head as boundary, was solved using the CHIEF method [36]. For a complete description and benchmark testing of BEMFA software, see Boonman et al. [37]. Using 3dsMax, we modified the bat’s gape by deforming the mesh by means of slight rotation and translation to within a range of natural postures with 10 different gape widths (the degree of opening was based on the observations carried out on the real bats), from each of which we calculated the sound-beam. In essence, parts of the inner mouth, such as tongue, palate, and inner cheeks, became acoustic radiators depending on the resonance at the given frequency, while the gape width represented variable apertures in elevation, from which these radiators can emit into the far field. We compared our results with a simple piston model with circular aperture whose radius or frequency can be varied (see supplementary materials for Additional file 10: equation and Additional file 11: Fig. S7).

For both the acoustic simulations and the measurements taken from live bats, gape width refers to the minimum distance from upper lip to lower lip during call emissions.

#### Statistical analysis

For the GLM analysis of the ICI, duration, mouth gape, and frequency (Fig. 1B, C), we used time bins of 0.05 s. We then examined whether there was an effect of the time (aligned to “0”) on any of the four parameters. We used a mixed effect generalized linear model (GLM) with ICI/duration/frequency/mouth gape as the explanatory parameter, time as the fixed factor, and the individual bats as a random effect. Random effects were set as intercepts (See also Additional file 2: Table S1).

## Supplementary Information

**Additional file 1: Figure S1.** Experimental set - up. The flight room was 5.5 x 4.5 x 2.5 m^3^ in size with acoustic foam on the walls and ceiling. Audio recordings were performed using 46 ultrasonic wide-band microphones. The bats were trained to search for and land on a platform where mealworms were offered. Twenty tracking cameras tracked the flight path and mouth gape of the bats. Red lighting depicts the IR light emitted from the cameras.

**Additional file 2: Table S1. ***P*-values of individual bats. *P* values of the statistical tests for the individual bats. Red values indicate significance below 0.05.

**Additional file 3: Figure S2.** Head direction vs. beam direction. The direction of the beam (Y), which was computed from the microphone array, was highly correlated with the direction of the head (X), which was computed from the tracking system. We could thus use the direction of the head as an approximation for the direction of the beam. The graph shows the average and STD for 5 bats. The Pearson correlation between the two was *R*=0.91.

**Additional file 4: Figure S3.** Head azimuth relative to direction of flight. The distribution of the head azimuth is shown for five bats (*n* = 5, mean and STD). X-axis represents flight direction. The peak at ~15 °s indicates that the bats directed their gaze slightly away from their direction of flight (‘0’ degrees) and towards the direction of turning. The peak was on the positive side because all bats used the same turning direction in the room.

**Additional file 5: Figure S4.** Mouth-opening speed. The bats maintained a steady speed when opening the mouth, across all ICIs. Mean ± SE, *n* = 5.

**Additional file 6: Figure S5.** Horizontal beam-width for different gapes and frequencies. The horizontal beam-width did not change when mouth gape width changed. 3D acoustic simulation of the horizontal beam-width as a function of the emission frequency for the two observed mouth gapes: 2.2 mm in green and 5.7 mm in brown. Horizontal and vertical dashed lines depict the bats’ peak and upper frequencies of the signal, respectively (40 kHz and 50 kHz, mean ± SE, *n* = 2).

**Additional file 7: Movie S1. ***P. kuhlii* flying in our flight room. The film was shot using a Phantom Miro camera at a frame rate of 800 fps. The opening of the mouth (a few milliseconds before the peak) and the moment of emission are depicted. The time period between two peak mouth openings is 42 ms on average in this film.

**Additional file 8: Figure S6.** Tracking system control. Bottom: the distance between two markers moving relative to each other – one is on the swinging pendulum and the other is on the base of the metronome. During the first part (left of the arrow) the metronome was stationary, and during the second part (right of the arrow) the metronome was moving. Red dashed line shows a smoothed filtering of the movement, and the error is estimated as the average distance between the smoothed and non-smoothed data. Top: the Y coordinate of the metronome's base reveals its movement.

**Additional file 9: Movie S2.** The performance of our tracking system when tracking the movement of a pendulum relative to the fixed base of the metronome.

**Additional file 10: Equation.** Circular aperture radiation / piston source model.

**Additional file 11: Figure S7.** The piston model -6 db beam-width for 65 kHz as a function of the aperture diameter.

## Data Availability

The datasets generated and analyzed during the current study are available on Mendeley Data 10.17632/8hwzr7jjmc.1 [38].
