# Supplementary figures and images for: Echolocating bats rapidly adjust their mouth gape to control spatial acquisition when scanning a target
Source: BMC Biol. 2022 Dec 17;20:282. doi: 10.1186/s12915-022-01487-w (PMC9758934; doi:10.1186/s12915-022-01487-w)

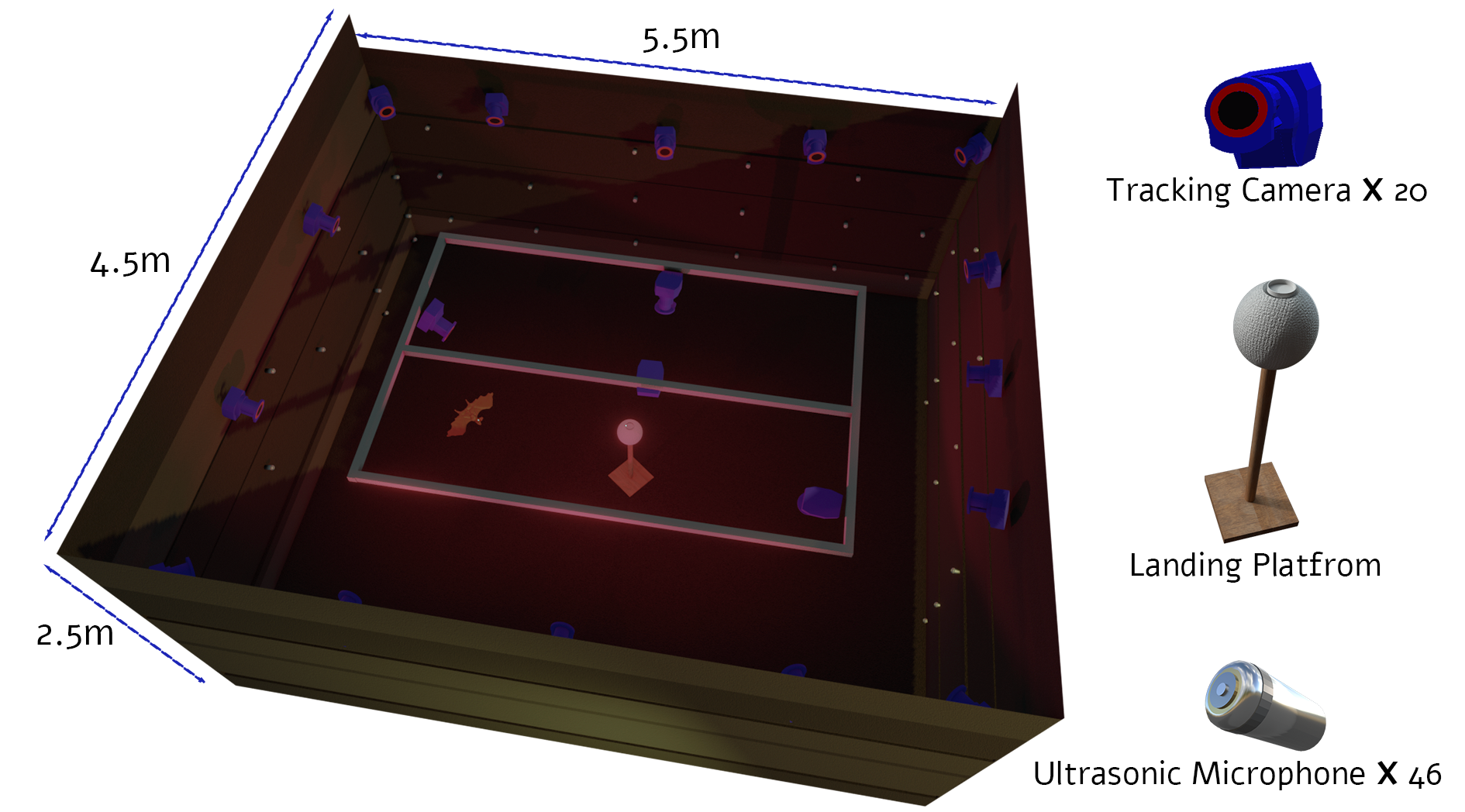

Supplement: Supplementary file 1 — Additional file 1: Figure S1. Experimental set - up. The flight room was 5.5 x 4.5 x 2.5 m3 in size with acoustic foam on the walls and ceiling. Audio recordings were performed using 46 ultrasonic wide-band microphones. The bats were trained to search for and land on a platform where mealworms were offered. Twenty tracking cameras tracked the flight path and mouth gape of the bats. Red lighting depicts the IR light emitted from the cameras. [file 12915_2022_1487_MOESM1_ESM.png]

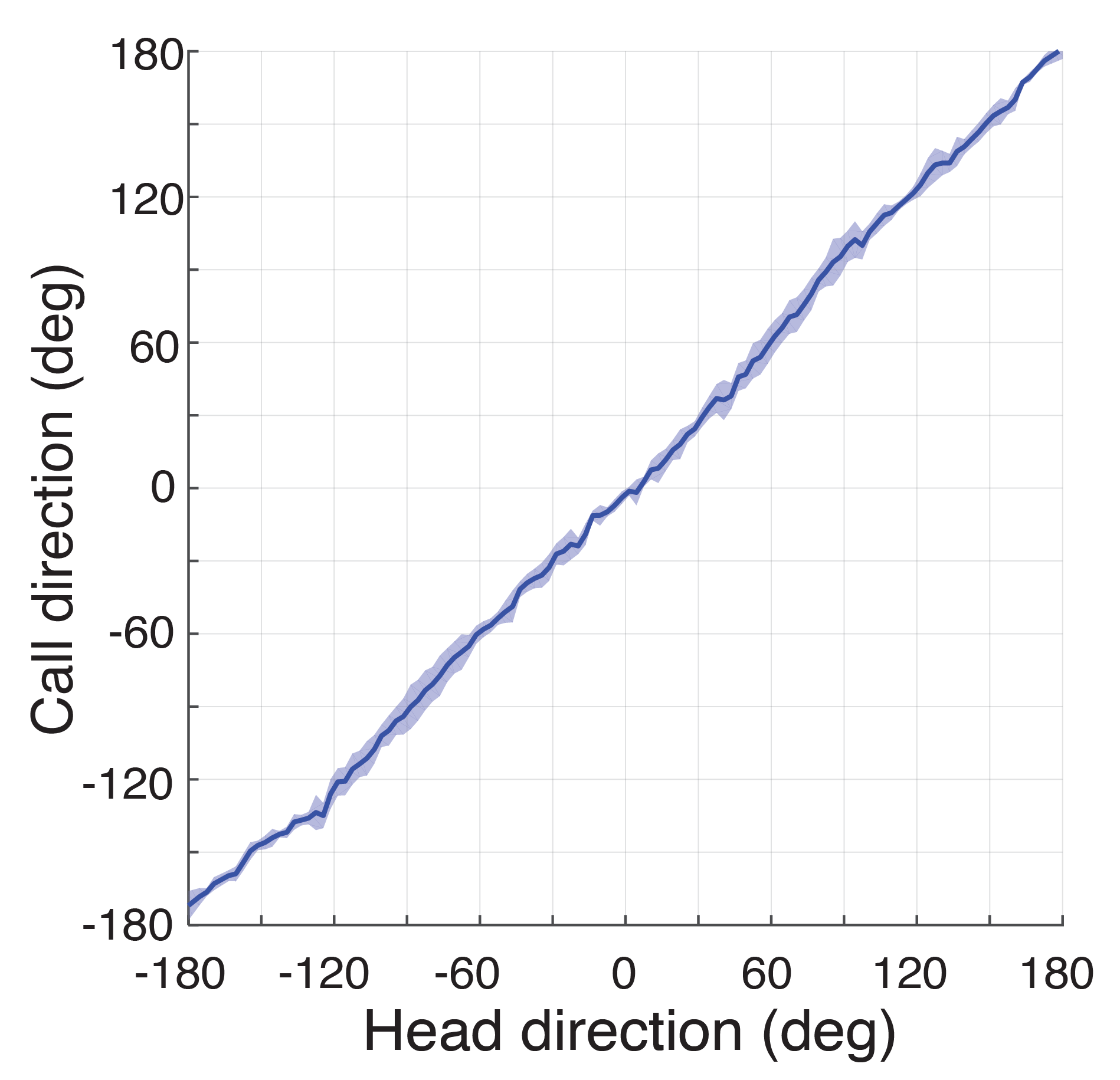

Supplement: Supplementary file 3 — Additional file 3: Figure S2. Head direction vs. beam direction. The direction of the beam (Y), which was computed from the microphone array, was highly correlated with the direction of the head (X), which was computed from the tracking system. We could thus use the direction of the head as an approximation for the direction of the beam. The graph shows the average and STD for 5 bats. The Pearson correlation between the two was R=0.91. [file 12915_2022_1487_MOESM3_ESM.png]

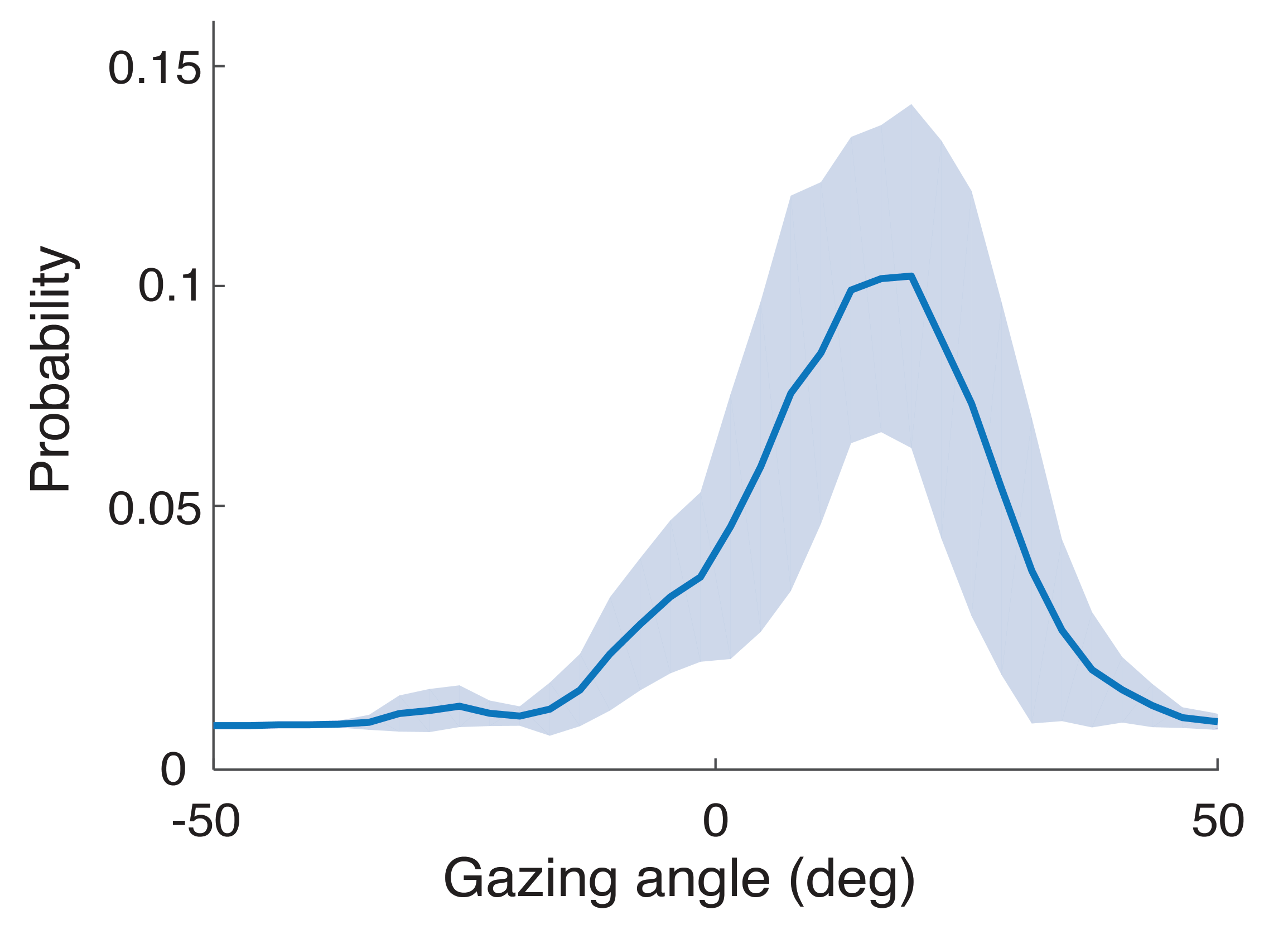

Supplement: Supplementary file 4 — Additional file 4: Figure S3. Head azimuth relative to direction of flight. The distribution of the head azimuth is shown for five bats (n = 5, mean and STD). X-axis represents flight direction. The peak at ~15 °s indicates that the bats directed their gaze slightly away from their direction of flight (‘0’ degrees) and towards the direction of turning. The peak was on the positive side because all bats used the same turning direction in the room. [file 12915_2022_1487_MOESM4_ESM.png]

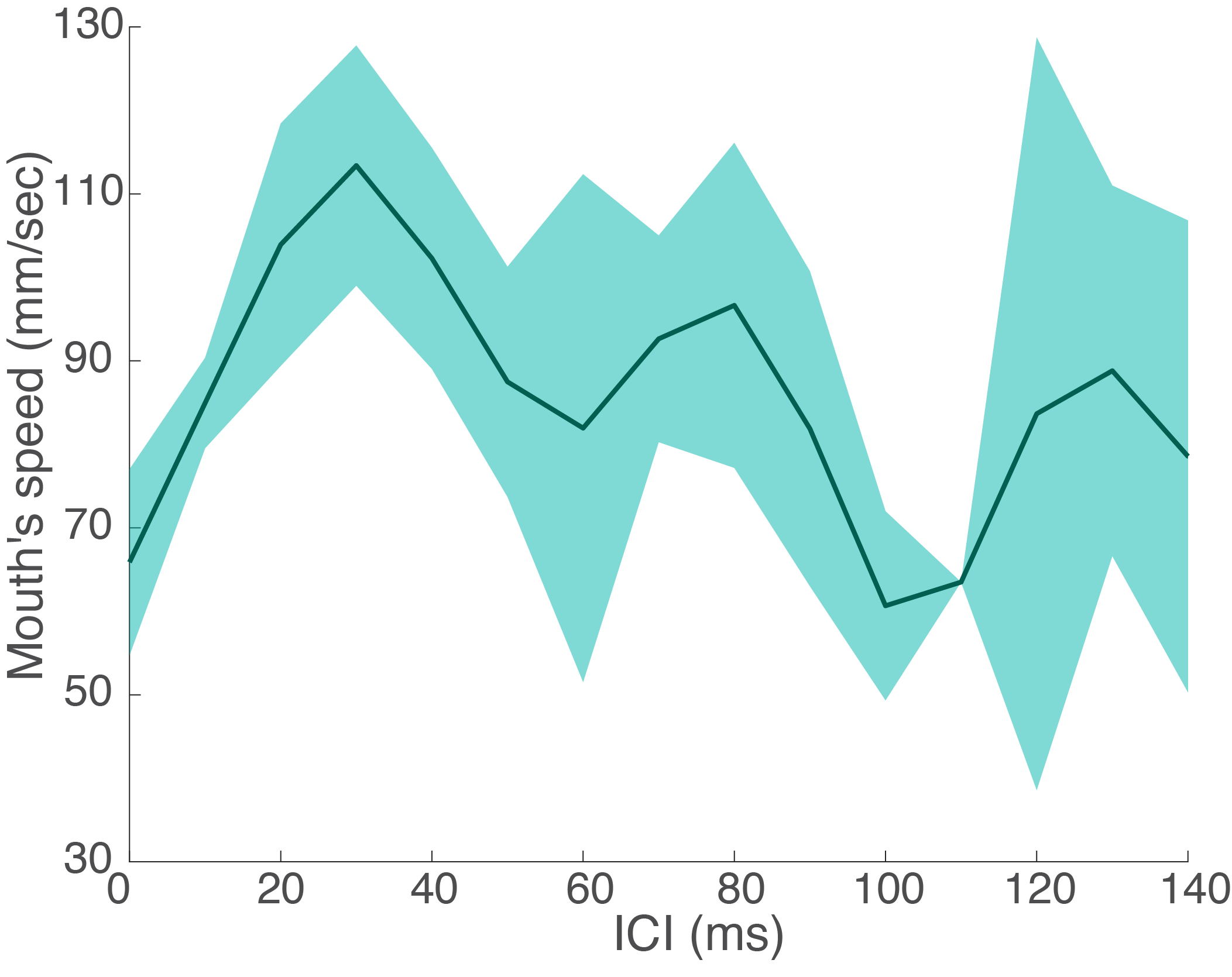

Supplement: Supplementary file 5 — Additional file 5: Figure S4. Mouth-opening speed. The bats maintained a steady speed when opening the mouth, across all ICIs. Mean ± SE, n = 5. [file 12915_2022_1487_MOESM5_ESM.png]

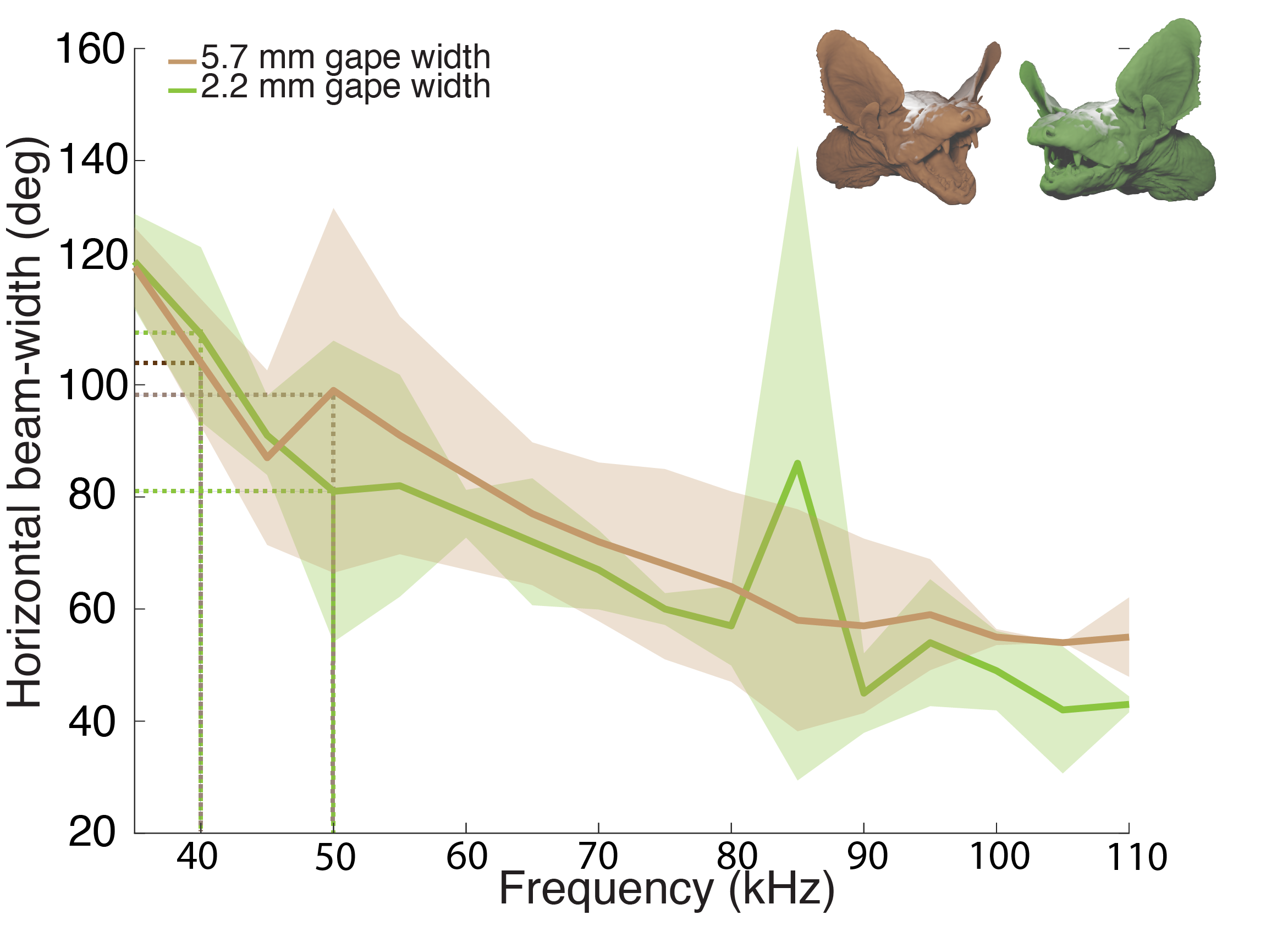

Supplement: Supplementary file 6 — Additional file 6: Figure S5. Horizontal beam-width for different gapes and frequencies. The horizontal beam-width did not change when mouth gape width changed. 3D acoustic simulation of the horizontal beam-width as a function of the emission frequency for the two observed mouth gapes: 2.2 mm in green and 5.7 mm in brown. Horizontal and vertical dashed lines depict the bats’ peak and upper frequencies of the signal, respectively (40 kHz and 50 kHz, mean ± SE, n = 2). [file 12915_2022_1487_MOESM6_ESM.png]

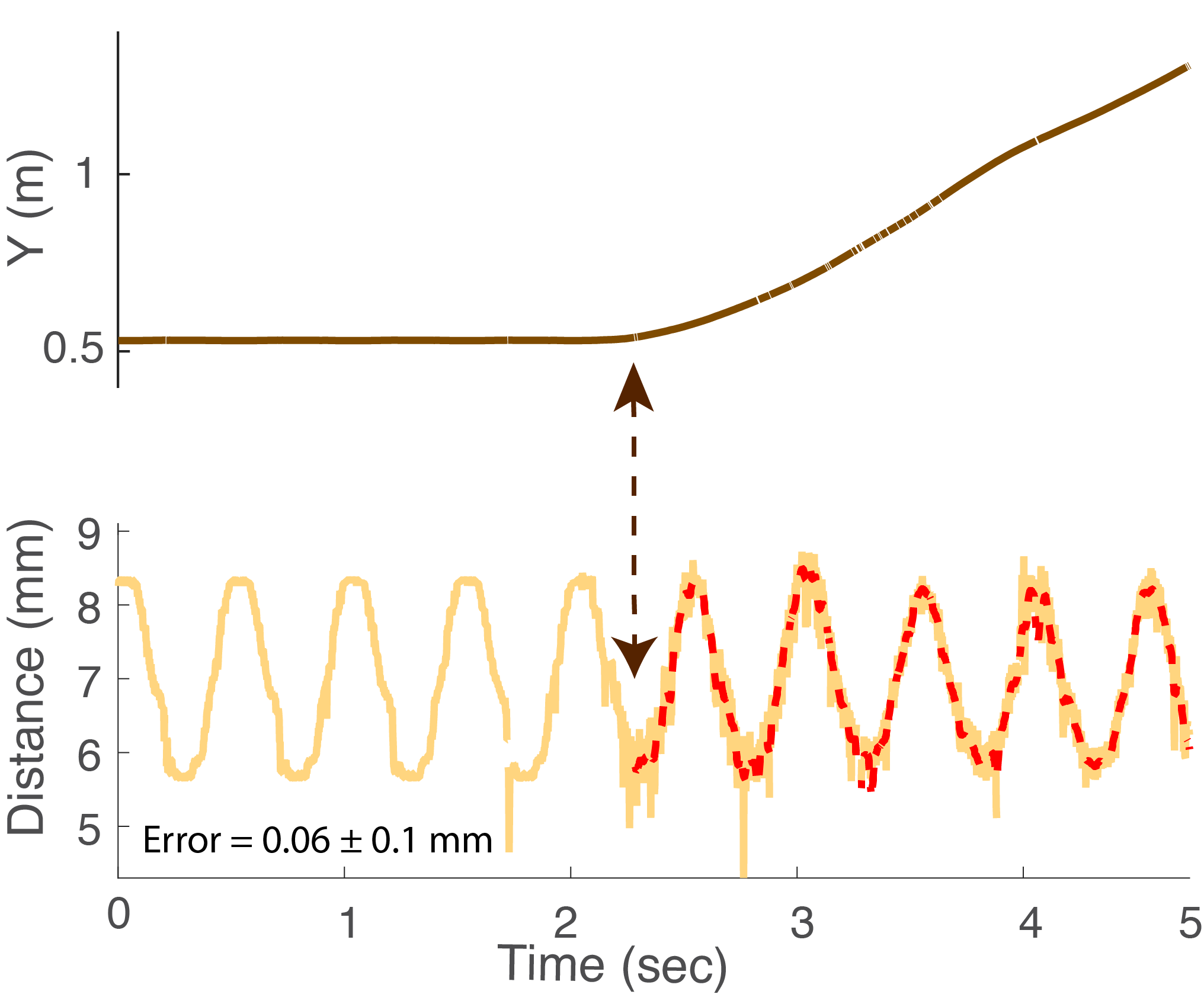

Supplement: Supplementary file 8 — Additional file 8: Figure S6. Tracking system control. Bottom: the distance between two markers moving relative to each other – one is on the swinging pendulum and the other is on the base of the metronome. During the first part (left of the arrow) the metronome was stationary, and during the second part (right of the arrow) the metronome was moving. Red dashed line shows a smoothed filtering of the movement, and the error is estimated as the average distance between the smoothed and non-smoothed data. Top: the Y coordinate of the metronome's base reveals its movement. [file 12915_2022_1487_MOESM8_ESM.png]

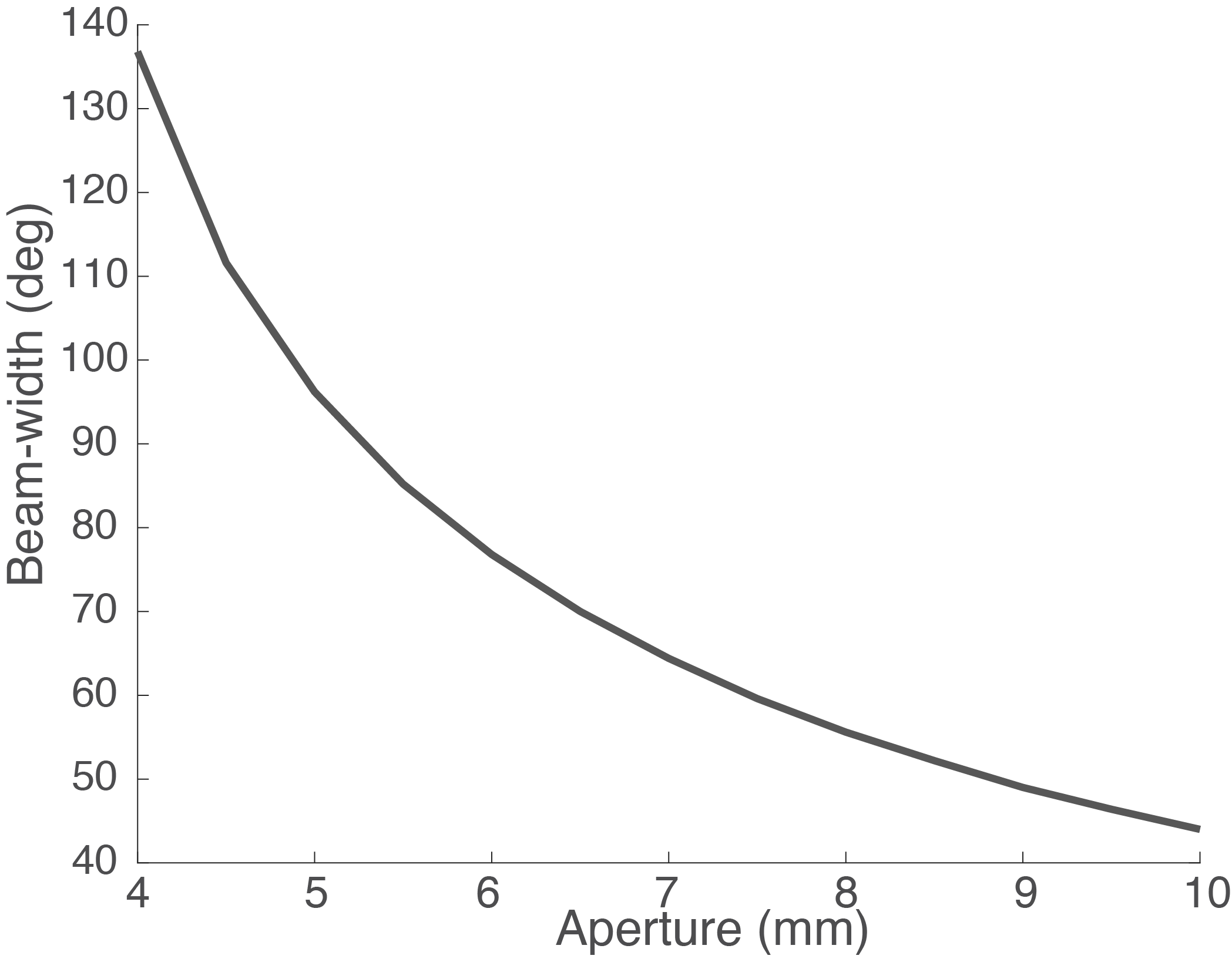

Supplement: Supplementary file 11 — Additional file 11: Figure S7. The piston model -6 db beam-width for 65 kHz as a function of the aperture diameter. [file 12915_2022_1487_MOESM11_ESM.png]
