# Supplementary material for: Echolocating bats rapidly adjust their mouth gape to control spatial acquisition when scanning a target
Source: BMC Biol. 2022 Dec 17;20:282. doi: 10.1186/s12915-022-01487-w (PMC9758934; doi:10.1186/s12915-022-01487-w)
Supplement: Supplementary file 2 — Additional file 2: Table S1. P-values of individual bats. P values of the statistical tests for the individual bats. Red values indicate significance below 0.05. [file 12915_2022_1487_MOESM2_ESM.docx]

| **Parameter** | **Test** | **Bat 1** | **Bat 2** | **Bat 3** | **Bat 4** | **Bat 5** |
| --- | --- | --- | --- | --- | --- | --- |
| Duration | mixed-effect GLM | 0.031 | 5.71E-05 | 0.003 | 0.023 | 0.001 |
| ICI | mixed-effect GLM | 8.96E-05 | 1.49E-07 | 3.66E-08 | 0.001 | 0.0001 |
| Frequency | mixed-effect GLM | 0.154 | 3.23E-07 | 1.97E-06 | 4.62E-07 | 1.45E-07 |
| Mouth Gape | mixed-effect GLM | 2.00E-06 | 1.04E-08 | 2.95E-11 | 6.28E-06 | 6.57E-07 |
