# Supplementary material for: Echolocating bats rapidly adjust their mouth gape to control spatial acquisition when scanning a target
Source: BMC Biol. 2022 Dec 17;20:282. doi: 10.1186/s12915-022-01487-w (PMC9758934; doi:10.1186/s12915-022-01487-w)
Supplement: Supplementary file 10 — Additional file 10: Equation. Circular aperture radiation / piston source model. [file 12915_2022_1487_MOESM10_ESM.docx]

**Circular aperture radiation / piston source model**

Intensity (in W/m^2) as a function of angle is given by:

$${I_{\theta}=Gain*\left| \frac{2 J_{1}(k_{0} a\sin\left( \theta\right))}{k_{0} a\sin\left( \theta\right)} \right|}^{2}$$

In which J_1_ denotes a first order Bessel function, $a$ denotes the radius of the aperture in meters, k_0_=2π/λ and θ is the angle of incidence in radians. Gain denotes the amplification due to emitter size and radiation efficiency with wavelength λ.

Gain = 4π A/λ^2^ in which A=πa^2^
